# Cyto/Biocompatibility of Dopamine Combined with the Antioxidant Grape Seed-Derived Polyphenol Compounds in Solid Lipid Nanoparticles

**DOI:** 10.3390/molecules26040916

**Published:** 2021-02-09

**Authors:** Adriana Trapani, Lorenzo Guerra, Filomena Corbo, Stefano Castellani, Enrico Sanna, Loredana Capobianco, Anna Grazia Monteduro, Daniela Erminia Manno, Delia Mandracchia, Sante Di Gioia, Massimo Conese

**Affiliations:** 1 Department of Pharmacy-Drug Sciences, University of Bari “Aldo Moro”, 70125 Bari, Italy; filomena.corbo@uniba.it; 2 Department of Biosciences, Biotechnologies and Biopharmaceutics, University of Bari “Aldo Moro”, 70125 Bari, Italy; lorenzo.guerra1@uniba.it; 3 Department of Biomedical Sciences and Human Oncology, University of Bari “Aldo Moro”, 70125 Bari, Italy; stefano.castellani@uniba.it; 4 Department of Life and Environmental Sciences, Section of Neuroscience and Anthropology, Faculty of Biology and Pharmacy, University of Cagliari, Cittadella Universitaria, 09042 Monserrato (Cagliari), Italy; esanna@unica.it; 5 Department of Biological and Environmental Sciences and Technologies, University of Salento, 73100 Lecce, Italy; loredana.capobianco@unisalento.it (L.C.); annagrazia.monteduro@unisalento.it (A.G.M.); daniela.manno@unisalento.it (D.E.M.); 6 Department of Molecular and Translational Medicine, University of Brescia, Viale Europa 11, 25123 Brescia, Italy; delia.mandracchia@unibs.it; 7 Department of Medical and Surgical Sciences, University of Foggia, 71122 Foggia, Italy; sante.digioia@unifg.it (S.D.G.); massimo.conese@unifg.it (M.C.)

**Keywords:** solid lipid nanoparticles, dopamine, grape seed-derived extract, physical stability, antioxidant activity, olfactory ensheathing, neuroblastoma SH-SY5Y cells

## Abstract

**Background:** The loss of nigrostriatal neurons containing dopamine (DA) together with the “mitochondrial dysfunction” in midbrain represent the two main causes related to the symptoms of Parkinson’s disease (PD). Hence, the aim of this investigation is to co-administer the missing DA and the antioxidant grape seed-derived proanthocyanidins (grape seed extract, GSE) in order to increase the levels of the neurotransmitter (which is unable to cross the Blood Brain Barrier) and reducing the oxidative stress (OS) related to PD, respectively. **Methods:** For this purpose, we chose Solid Lipid Nanoparticles (SLN), because they have been already proven to increase DA uptake in the brain. DA-SLN adsorbing GSE (GSE/DA-SLN) were formulated and subjected to physico-chemical characterization, and their cytocompatibility and protection against OS were examined. **Results:** GSE was found on SLN surface and release studies evidenced the efficiency of GSE in preventing DA autoxidation. Furthermore, SLN showed high mucoadhesive strength and were found not cytotoxic to both primary Olfactory Ensheathing and neuroblastoma SH-SY5Y cells by MTT test. Co-administration of GSE/DA-SLN and the OS-inducing neurotoxin 6-hydroxydopamine (100 μM) resulted in an increase of SH-SY5Y cell viability. **Conclusions:** Hence, SLN formulations containing DA and GSE may constitute interesting candidates for non-invasive nose-to-brain delivery.

## 1. Introduction 

Parkinson’s disease (PD) is considered a movement disorder that affects about 1–2% of the population above 65 years with a prevalent incidence in older adults of male gender [1]. The main neuropathological evidence of PD is represented by the loss of dopaminergic neurons in the Substantia Nigra and the presence of α-synuclein-containing Lewy bodies within neurons [2]. In order to limit the PD symptoms [3], the so-called “dopamine (DA) replacement strategy” is currently followed by administering levodopa (L-Dopa) that constitutes the reference drug in anti-PD therapy [4]. L-Dopa can overcome the blood-brain-barrier (BBB) thanks to an active transport system and in the brain, it is converted into the neurotransmitter DA by L-Dopa-decarboxylase mediated decarboxylation, whereas DA cannot cross the BBB due to its unfavorable physico-chemical features [5].

Although the etiology and PD pathogenesis still remain to be fully clarified, in past decades some steps forward occurred regarding the knowledge of the molecular basis underlying the DAergic neuron death in this neurodegenerative disease. In addition to the α-synuclein (and its aggregates) toxicity, increasing evidence suggests that the so-called “mitochondrial dysfunction and oxidative stress” and the “neuroinflammation” mechanisms are involved in PD pathogenesis [6,7]. In particular, dysregulation in mitochondrial dynamics, inhibition of the electron transport chain and increased reactive oxygen species (ROS) production have been related to the death of DAergic neurons [6,7].

A further advance made for the treatment of neurodegenerative diseases, including PD, is represented by the observation that appropriately tailored nanocarriers may be able to overcome the challenging obstacle represented by the BBB, exploiting the physiological mechanisms of transport [8,9]. It should be considered, indeed, that, although the BBB, in the presence of neurodegenerative disease, as PD and Alzheimer’s disease, is altered in structure and permeable enough, it still limits the brain penetration of neurotherapeutics and brain imaging agents [10]. Nowadays, it is possible to prepare several suitably designed nanocarriers to solve many problems including the BBB. Thus, DA-loaded-nanoparticles or -micelles or -liposomes can increase the stability of the neurotransmitter and can allow its sustained release and avoid its peripheral metabolism. For instance, the approach based on laser ablation allows the synthesis of ultrasmall nanomaterials (<100 nm) with several applications, including the crossing of the BBB, just owing to their small size [11,12].

In addition to those mentioned above, another interesting and non-invasive approach for bypassing the BBB is intranasal drug administration by which brain delivery of therapeutics is achieved by transport of molecules from the nasal cavity to the brain parenchyma along the olfactory or trigeminal nerves [6,13,14,15]. It is noteworthy that the intranasal administration route can be, sometimes, an alternative to the oral one, which matches patient compliance and the approval of the pharmaceutical industry [16].

Moving from this background, the purpose of the present work was to evaluate the potential of the nose-to-brain delivery of antioxidant agents as polyphenol compounds and the neurotransmitter DA, both co-administered in Solid Lipid Nanoparticles (SLN) intended as a novel delivery system to achieve improved therapeutic efficacy and reduced side effects in the treatment of PD. The rationale behind this choice lies on the hypothesis that the development of new antioxidant-based therapies may limit the contribution of oxidative stress (OS) in determining the death of DAergic neurons. This hypothesis is supported by recent findings showing that lipid peroxidation suppression of methyl linoleate dispersed in Triton X-100 micelles can be achieved by using DA or L-Dopa in the presence of an antioxidant agent as 2,2,5,7,8-pentamethyl-6-hydroxychroman, or PMHC, an analog of α-tocopherol [17]. Moreover, it has been also described that the co-administration of L-Dopa and resveratrol to 1-methyl 4-phenyl 1,2,3,6-tetrahydro-pyridine (MPTP) treated mouse model of PD resulted in a marked reduction of L-Dopa side effects in long-term therapy [18]. We chose to investigate SLN in this study since they are characterized by several advantages including enhanced safety, stability and controlled drug release, and can be prepared on large scale. Moreover, apart from cyclodextrins [19] and polymeric micelles [20], SLN are nanocarriers widely employed to improve the formulation of hydrophobic drugs and they can be administered in several ways, including through the lymphatic system which is directly connected with brain tissues and cerebrospinal fluid [13,14].

**Figure 1:**
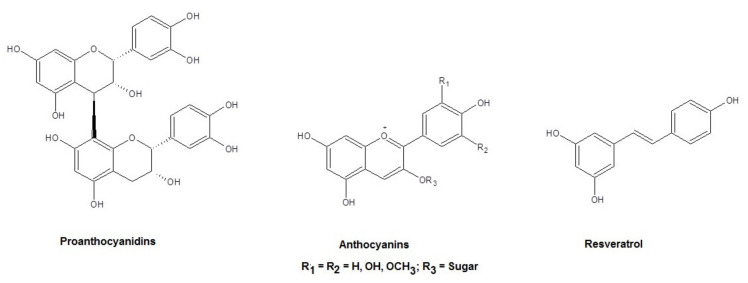
Chemical structures of the main polyphenol compounds represented in grape seed extract.

The SLN prepared in the present work are based on the self-emulsifying lipid Gelucire^®^ 50/13 which is a mixture of PEG-esters (stearoyl polyoxyl-32 glycerides), a small glyceride fraction and free PEG chains. Therefore, the resulting SLN may be considered PEGylated SLN. The selection of this lipid matrix was motivated on the basis of our recent works where we showed that such self-emulsifying lipid-based SLN are able to efficiently load hydrophilic active principles other than the hydrophobic ones [21,22,23]. It should be considered, indeed, that both the polyphenol antioxidant agents and the neurotransmitter DA included in this study are hydrophilic compounds. In particular, we chose polyphenol compounds occurring in grape seed-derived extract (GSE) in which proanthocyanidins are the most represented compounds and anthocyanins and resveratrol to a low extent (Figure 1). As far as the bioactivity of polyphenol compounds from grapes is concerned, the most relevant and investigated is the antioxidant compound. Apart from this effect, further biological activities have been recognized for grape seed-derived substances such as anti-inflammatory, anti-cancer, anti-microbial and anti-aging activities [24].

On the whole, the aim of the present work was to evaluate the co-administration of GSE/DA–loaded-SLN by nasal route as a disease-modifying approach for PD treatment, allowing not only the BBB to be overcome but also the limitation of the OS and neuroinflammation mechanisms. To the best of our knowledge, this is the first example of contemporary administration of DA and antioxidant agents (GSE) in a unique pharmaceutical dosage form intended for nose-to-brain delivery. In this paper, we report the preparation of the required GSE/DA–loaded SLN and their physico-chemical characterization as well as the in vitro release studies and the evaluation of the antioxidant effect. Finally, the evaluation of cell viability in primary Olfactory Ensheathing cells (OECs) and neuroblastoma SH-SY5Y cells were also determined, and the obtained results are reported below and discussed.

## 2. Results

### 2.1. Preparation and Physico-Chemical Characterization of SLN

Our previous works showed that both DA and GSE can be singularly encapsulated in Gelucire^®^ 50/13 based SLN, by using the melt-emulsification method [21,22,23,25]. To supply both DA and GSE in a unique formulation consisting of Gelucire^®^ 50/13 based SLN, DA-SLN were preformed and then adsorption of GSE on the surface occurred. The main physico-chemical properties including particle size, polydispersity index, zeta potential and encapsulation efficiency (E.E.) of SLN herein studied are shown in Table 1, where plain SLN and GSE-SLN (i.e., plain SLN adsorbing GSE on their surface) formulations are also included for comparative purposes. As can be seen from Table 1, the use of diluted acetic acid for SLN formulation caused a pH value decrease (around pH = 3) of the final suspension, but, as already reported [22], thanks to this pH value intact DA can be preserved.

The comparison of the particle size of GSE/DA-SLN with those of other formulations herein studied showed that the differences were statistically significant only by comparing GSE-SLN with GSE/DA-SLN (*p* < 0.01) or GSE-SLN with DA-SLN (*p* < 0.05), whereas in the remaining cases they resulted not significant. Hence, the mean diameter of the starting DA-SLN was 171 ± 6 nm but underwent a not statistically significant increase when incubated with GSE solution to provide DA-SLN adsorbing GSE (i.e., GSE/DA-SLN) with a mean diameter of 184 ± 34 nm. The low PDI values for DA-SLN and GSE-SLN suggest that these formulations possess a moderate uniformity in size, while a slightly broader size distribution should be related to GSE/DA-SLN being the PDI value of 0.32 ± 0.07. The zeta potential value of GSE/DA-SLN was close to the neutrality as occurs for the DA-loaded SLN (i.e., DA-SLN). Hence, the zeta potential of these last particles was not affected when incubation with GSE occurs.

**Figure 2:**
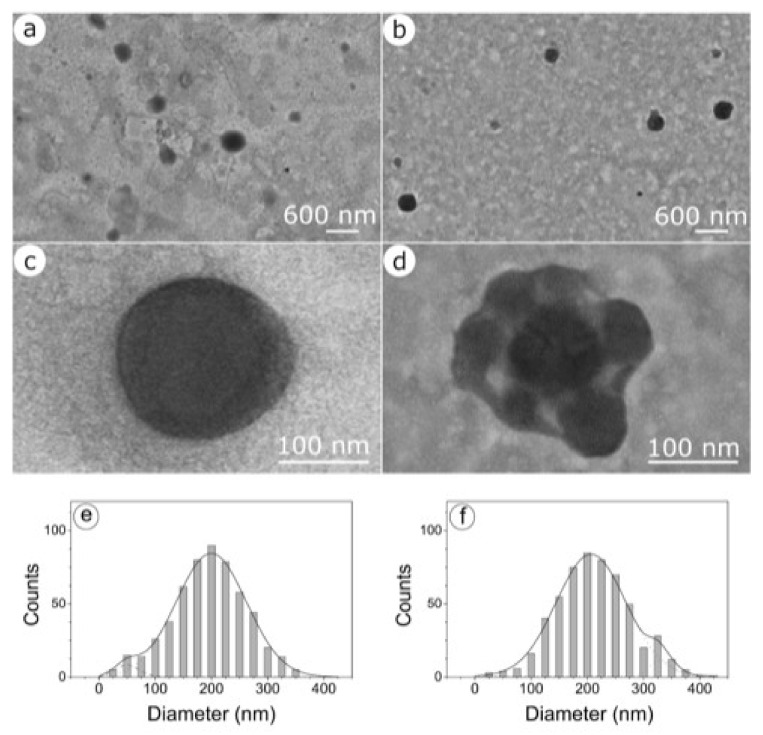
TEM microphotographs of DA-SLN (panel (**a**,**c**,**e**)) and GSE/DA SLN (panel (**b**,**d**,**f**)).

As for the encapsulation efficiency (E.E.%), in this work the quantitative determination of both the two biologically active substances (DA and GSE) was carried out by HPLC analysis (Section 4.2), omitting the colorimetric assay previously used for GSE determination [25]. From data reported in Table 1, it appears that both the DA and the GSE content occurring in GSE/DA-SLN were not different in a statistically significant manner from those found in DA-SLN and GSE-SLN, respectively. Furthermore, thanks to the vitrification process, it was possible to observe by cryo-TEM the morphology of DA-SLN and GSE/DA-SLN and to carry out an accurate dimensional analysis (Figure 2).

As shown in Figure 2a,b, both DA-SLN and GSE/DA-SLN display an almost spherical shape. Unlike DA-SLN, whose nanoparticles remain isolated (Figure 2c), GSE/DA-SLN exhibit an external layer surrounding particles that sometimes aggregate, forming complexes in which small particles gather outside the main nanoparticle (Figure 2d). Moreover, DA-SLN showed a bimodal particle distribution (Figure 2e) with most of the nanoparticles distribute around the value of 200 nm and a standard deviation of 60 nm and a low concentration of nanoparticles having a mean diameter around 50 nm and a standard deviation of 15 nm. GSE/DA-SLN also showed a bimodal particle distribution (Figure 2f) with most of the nanoparticles distributed around the value of 210 nm and a standard deviation of 60 nm and a low concentration of nanoparticles at mean particle size equal to 340 nm and standard deviation of 6 nm.

Therefore, it appears that GSE adsorption shifts the size distribution of nanocarriers towards larger values.

Indirect information on adsorption is given by the ability of nanoparticles to cling to form cluster-shaped complexes.

**Figure 3:**
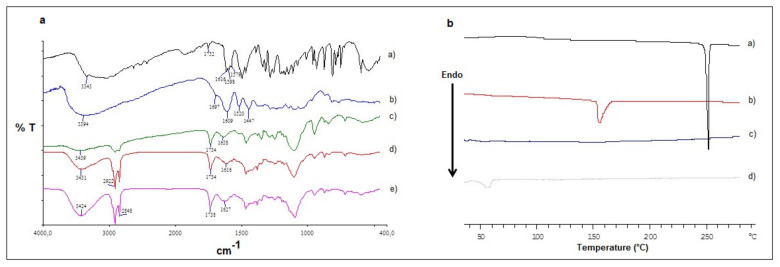
Panel (**a**): FT-IR spectra of pure DA (**a**), pure GSE (**b**), DA-SLN (**c**), GSE/DA-SLN (**d**) and GSE-SLN (**e**). Panel (**b**): DSC thermograms of pure DA (**a**), pure GSE (**b**), DA-SLN (**c**) and GSE/DA-SLN (**d**).

Moreover, information on the solid state of the SLN prepared was obtained by FT-IR spectroscopy and Differential Scanning Calorimetry (DSC). In Figure 3, the FT–IR spectra of pure DA, pure GSE, DA-SLN, GSE-SLN and GSE/DA-SLN as well as the thermograms of pure DA, pure GSE, DA-SLN and GSE/DA-SLN are reported.

In particular, in the FT-IR spectrum of GSE/DA-SLN, three characteristic absorption bands were noted at 3431 cm^−1^, 1734 cm^−1^ and 1616 cm^−1^ which were also present in the spectra of the other corresponding SLN formulations (i.e., DA-SLN and GSE-SLN) even though slightly shifted. It should be noted that the absorption bands at 3431 cm^−1^, and 1734 cm^−1^ could be ascribed to partially hydrated Gelucire^®^ 50/13, while in all these spectra the characteristic peaks attributable to pure DA and pure GSE at 1616–1579 cm^−1^ and at 1609 cm^−1^, respectively, were not distinctly detected [26,27]. In the DSC thermogram of GSE/DA-SLN the endothermic peaks attributable to the melting of pure DA and pure GSE (at 250 °C and 160 °C, respectively) were not detected. Instead, a broad peak was observed at about 55 °C attributable to pure Gelucire^®^ 50/13 lipid [28]. Hence, the DSC profile of GSE/DA-SLN is similar to that of the pure lipid matrix.

### 2.2. Physical Stability of GSE/DA-SLN

**Figure 4:**
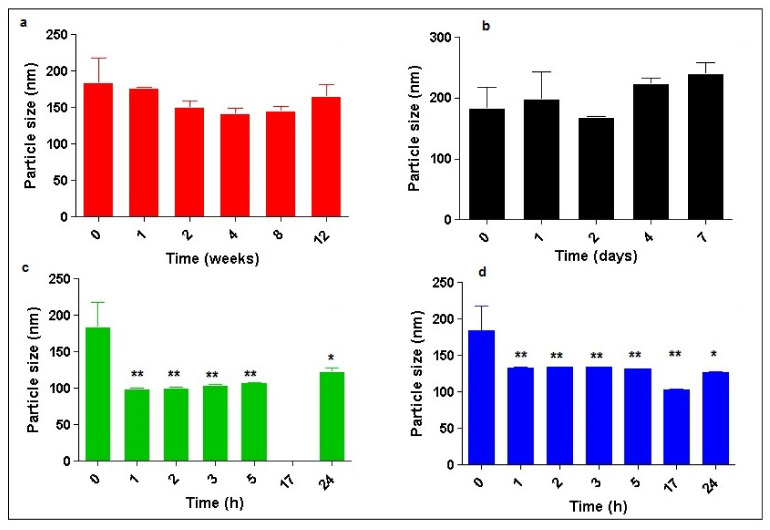
Particle size variations after storage of GSE/DA-SLN at 4 °C (panel (**a**)) or at 25 °C (panel (**b**)) or at 37 °C in PBS pH 7.4 (panel (**c**)) and at 37 °C in SNF (panel (**d**)); statistical significance: * *p* < 0.05, ** *p* < 0.01 vs. the starting particle size value.

The physical stability of GSE/DA-SLN was evaluated by measuring the particle size of this formulation after storage at 4 °C for 12 weeks or at 25 °C for one week as well as after incubation for 24 h at 37 °C in PBS pH 7.4 or at 37 °C in Simulated Nasal Fluid (SNF) and the observed results are shown in Figure 4. As seen, storage at 4 °C for some weeks determined a decrease in particle size, even though it was not significant from a statistical point of view (Figure 4a). On the other hand, after two days of storage at 25 °C, a statistically significant decrease in particle size resulted, while storage for more than two days led to a not significant increase in size of the particles which resulted in slightly higher than 200 nm (Figure 4b). Interestingly, storage at 37 °C in PBS pH 7.4 or in SNF for 24 h gave a significant decrease in the particle size (Figure 4c,d, respectively). In both cases, this storage produced particles in the range from about 100 nm to 150 nm. However, a marked difference was observed between the storage in PBS pH 7.4 and that in SNF after 17 h of incubation. In fact, after this time it was noted that the suspension in PBS pH 7.4 turned black, while such a change did not occur for the suspension in SNF.

### 2.3. Mucoadhesion of GSE/DA-SLN in Simulated Nasal Fluid 

**Figure 5:**
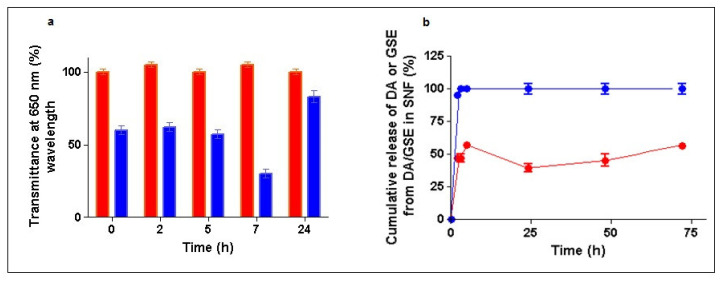
Panel (**a**): Mucoadhesive properties in simulated nasal fluid (SNF) of GSE/DA-SLN (blue bars) with hydroxyethylcellulose (HEC) (red bars) taken as positive control. Panel (**b**): In vitro release of dopamine (red line) and grape seed extract (GSE) (blue line) from GSE/DA-SLN in SNF in the presence of esterases at 37 °C.

The in vitro mucoadhesive properties of GSE/DA-SLN were evaluated by turbidimetric measurements after their incubation in SNF [29] for 24 h. Changes in transmittance at 650 nm wavelength were recorded during 24 h of SLN exposure to SNF, comparing the results with those of Hydroxyethyl cellulose (HEC) adopted as a positive control [30] instead of Carbopol 940 which, on the other hand, could not be employed due to its precipitation in SNF. No change in color of the tested formulations throughout the study was visually revealed which suggests that no chemical (oxidative and hydrolytic) degradation occured. As shown in Figure 5a, GSE/DA-SLN exhibited the most relevant reduction decrease in transmittance after 7 h of incubation time, resulting in a statistically significant difference compared with the control (*p* < 0.001 vs. HEC, respectively) suggesting high mucoadhesive properties for this nanostructured formulation.

### 2.4. In Vitro Release Studies of GSE/DA-SLN 

In vitro release of GSE and DA from GSE/DA-SLN was studied in SNF containing esterases and the corresponding delivery profile is shown in Figure 5b. As seen, GSE was released from GSE/DA-SLN in a prompt and complete manner within two hours, displaying a clear burst effect. In contrast, DA was delivered in a slower fashion reaching about 60% within the first five hours afterwards, and the levels of the neurotransmitter decreased up to 45% within 24 h followed by a slow increase up to 60% of DA released in the next two days. It is noteworthy that, upon such release test conditions, DA did not undergo any degradation and remained intact for all of the time of the study (72 h). This behavior could be due to the simultaneous presence of the antioxidant GSE.

### 2.5. Antioxidant Activity of SLN Prepared

The prepared SLN were evaluated for their antioxidant activity by using a slightly modified spectrophotometric method based on the use of 2,2-diphenyl-1-picrylhydrazyl (DPPH) free radical, enabling us to assess the ability of chemical substances to act as free radical scavengers or hydrogen donors and the obtained results, referring to pure GSE selected as control (i.e., antioxidant activity equal to 100%) are reported in Table 2 [31]. As shown, the pure Gelucire^®^ 50/13 lipid matrix displayed antioxidant activity in the amount of about 50% of pure GSE and this percentage increased to 72.8 ± 5.3 when the lipid matrix was nanostructured to give plain SLN. It is noteworthy that encapsulation of DA in SLN, providing DA-SLN, brought about a reduction of antioxidant activity of about 18%. Indeed, the adsorption of GSE onto SLN, giving rise to GSE-SLN, caused a reduction of antioxidant activity compared to that of pure GSE of about 16%. Instead, the adsorption of GSE on the surface of DA-SLN providing GSE/DA-SLN gave an increase in antioxidant activity of about 24% (from 54.7 ± 2.5 to 78.6 ± 3.6) and, therefore, the antioxidant activity of GSE/DA-SLN can be considered satisfactory enough and comparable to that of GSE-SLN.

### 2.6. In Vitro Cytotoxicity Test 

**Figure 6:**
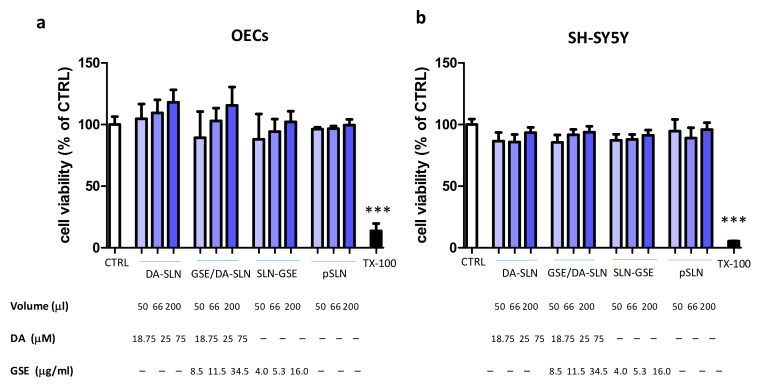
Cytocompatibility of SLN containing DA, GSE or DA and GSE. OECs (**a**) and SH-SY5Y cells (**b**) were challenged with DA-SLN, GSE/DA-SLN and SLN-GSE at the indicated volumes and concentrations of DA or GSE for 24 h. Plain SLN (pSLN) were used at equivalent volumes. Cells were then assayed for vitality by the MTT assay. Control (CTRL) cells are untreated cells (100% of vitality), whereas TX-100 (0.1% Triton X-100) denotes positive controls. Data are expressed as average ± SD of two experiments carried out each in six wells. *** *p* < 0.0001, TX-100 versus all other conditions.

Considering the olfactory transport of nanoparticles to the brain, the compatibility of SLN formulations was studied on OECs and SH-SY5Y neuroblastoma cells. All formulations tested by the (3-(4,5-Dimethylthiazol-2-yl)-2,5-Diphenyltetrazolium Bromide, MTT) assay (DA-SLN, GSE/DA-SLN, SLN-GSE) were not cytotoxic to OECs in comparison with negative controls (CTRL) and Plain-SLN (Figure 6a). Cytocompatibility was also observed when SH-SY5Y cells were incubated with the three formulations, which did not cause any cell toxicity in comparison to negative controls and Plain-SLN (Figure 6b).

### 2.7. 6-OHDA Cytotoxicity Model

**Figure 7:**
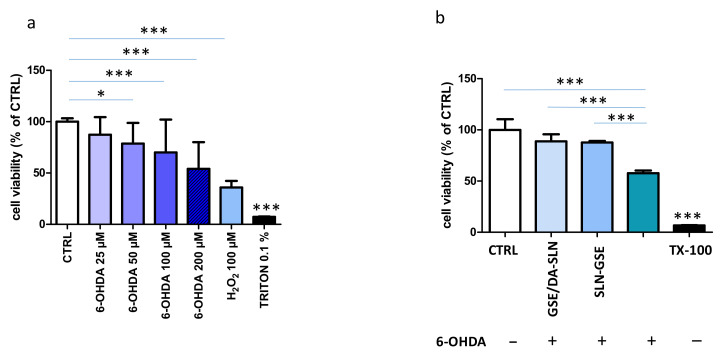
Cytoprotective effect of SLN containing DA or GSE on SH-SY5Y cells. (**a**) SH-SY5Y cells were challenged with different concentrations of 6-OHDA or H_2_O_2_ (100 μM) as indicated for 24 h. Cells were then assayed for vitality by the MTT assay. Control (CTRL) cells are untreated cells (100% of vitality), whereas TX-100 (0.1% Triton X-100) denotes positive controls. * *p* < 0.05; *** *p* < 0.0001. (**b**) SH-SY5Y cells were challenged with 6-OHDA (100 μM) and at the same time with either GSE/DA SLN (75 mM DA and 34.5 mg/mL GSE) or SLN-GSE (16 μg/mL). Untreated cells (CTRL, 100% of vitality) and TX-100 (0.1% Triton X-100) were used as negative and positive controls, respectively. Data are expressed as average ± SD of two experiments carried out each in six wells. *** *p* < 0.0001.

6-hydroxydopamine (6-OHDA) is widely used to produce animal models of Parkinson’s disease and is indeed a toxin selective for catecholaminergic cells [32]. 6-OHDA determined a reduction in SH-SY5Y cell viability in a dose-dependent manner, an effect reproduced by incubation with H_2_O_2_ (Figure 7a). We chose for further experiments to use the 6-OHDA concentration of 100 μM, which had sub-optimal cytotoxicity. In order to comprehend whether SLN studied in this work were cytoprotective, they were administered at the same moment of 6-OHDA. This set of experiments clearly shows that GSE/DA-SLN determined an increase in cell viability as compared with 6-OHDA-treated only cells (Figure 7b). Interestingly, GSE-SLN also had the same effect.

## 3. Discussion 

Among the novel approaches investigated for PD treatment, most interest has been focused in recent years on the development of DA-loaded nanocarriers, since they may be able to cross the BBB enabling a sustained delivery of the neurotransmitter to the brain [8,9]. In this context, the present work aimed at evaluating the feasibility of the co-administration by intranasal route of DA together with antioxidant agents such as those occurring in GSE, both administered in a unique delivery system represented by SLN, in order to achieve not only the overcoming of the BBB in a non-invasive manner but also the limitation of the OS and neuroinflammation mechanisms. Indeed, it should be expected that the antioxidant activity of GSE can preserve from progressive neurological damages typical of PD, thanks to its well-recognized role of scavenger towards free radical species [24]. For this purpose, we prepared DA-SLN adsorbing GSE on the surface to give GSE/DA–loaded SLN and their physico-chemical features were investigated. As mentioned above, the particle size of GSE/DA-SLN was not significantly different from the DA-SLN one and it means that the adsorption of GSE on the surface of DA-SLN did not produce marked changes in particle dimensions. It is noteworthy that after the GSE adsorption, the mean diameter of the particles still was around 200 nm, a value regarded as suitable for nose-to-brain delivery [33,34]. The adsorption of GSE on DA-SLN to give GSE/DA-SLN also did not change the surface charge close to the neutrality of these particles. The preparative procedure, consisting in the adsorption of GSE on the surface of DA-SLN, did not change significantly the E.E.% of the neurotransmitter and polyphenol compounds compared to those observed in DA-SLN and GSE-SLN, respectively. The E.E.% of DA in GSE/DA-SLN resulting in about 15% can be considered satisfactory considering that the neurotransmitter is a very potent substance. The amounts of DA in the GSE/DA-SLN (2.9–11.5 µg/mL corresponding to 18.75–75 μM, see Figure 6) appears to be within the pharmacological range. In fact, in a recent study, Tang et al. [35] showed that DA-carrying nanoparticles were effective in alleviating the apomorphine-induced turning behavior in a rat model of PD at the dose of ~7.4 mg/mL. In addition, intranasal administration of unconjugated DA has been thoroughly investigated in a variety of rat models, including the 6-OHDA model of PD, and shown to increase brain DA levels and produce a multitude of behavioral effects at doses that, based on the optimal volume for intranasal administration in rats, i.e., 50 µL [36], translate to concentrations ranging between 0.3 and 6 mg DA/mL [37,38,39,40]; this is higher, but it is expected that in such unconjugated form, the rate of absorption of DA in the nasal cavity is much lower, with enhanced susceptibility to local metabolism, than when DA is carried by nanoparticles. However, we have previously reported that the E.E.% of DA can be markedly increased if the neurotransmitter is encapsulated in Gelucire^®^ 50/13 based SLN in the presence of chitosan derivatives as coating limiting the immature leakage of DA occurring during sample manipulations in absence of chitosan coating [23]. Hence, it is expected that a marked increase in DA E.E% should occur whether the adsorption of GSE is performed on the surface of DA-SLN. In such a case, the presence of GSE should allow not only the positive effect on DA E.E.% but also on the mucoadhesive properties of the resulting nanocarrier [41,42]. For GSE/DA-SLN, cryo-TEM visualization allowed to us to discover a sort of an external coating and, interestingly, the diameter of the nanoparticles under the cryo-TEM microscope, taking into account the wide standard deviation, is in good agreement with the one determined by traditional light scattering.

Furthermore, the solid-state study on GSE/DA-SLN based on FT-IR spectroscopy and thermal analysis (DSC) were performed in order to gain information on the internal structure of neurotransmitter and polyphenol compounds in these SLN. From the results reported in Figure 3, it is apparent that both the FT-IR spectrum and the DSC thermogram of GSE/DA-SLN are similar to those of the pure lipid matrix, respectively. Moreover, both the characteristic absorption peaks in the FT-IR spectrum attributable to pure DA and pure GSE as well as the endothermic peaks in the DSC thermogram attributable to these pure substances were not detected. Such findings are indicative of a marked reduction in the crystalline state of both the neurotransmitter and polyphenol compounds, leading to an amorphous state of these two substances when combined in GSE/DA-SLN.

The low zeta potential value exhibited by GSE/DA-SLN (i.e., −2.7 ± 0.2, Table 1) suggests that these nanocarriers may have physical stability issues and it made mandatory a more in-depth study. However, it should be taken into account that DA may be decomposed in the presence of molecular oxygen by a spontaneous autoxidation reaction under neutral/alkaline conditions leading to a key intermediate formation denoted as aminochrome, and then to the synthesis of grey-black polymer compounds (e.g., neuromelanins) [23,43]. Protection of DA towards autoxidation reaction can be obtained to some extent by encapsulation of the neurotransmitter in nanocarriers as liposomes and SLN [23,43]. Hence, the physical stability was evaluated not only by monitoring the particle size of GSE/DA-SLN but also by observing possible color changes and/or formation of grey-black precipitates from GSE/DA-SLN suspension, this last being indicative that the DA autoxidation reaction is in progress [23,43]. As reported in Section 2.2, storage of a dispersion of GSE/DA-SLN at 4 °C for 12 weeks or at 25 °C for one week produced no significant change in particle size. Instead, storage at 37 °C in PBS pH 7.4 or in SNF for 24 h gave a significant decrease in particle size (Figure 4c,d, respectively), leading to particles in the range from about 100 nm to 150 nm. These interesting results may account for the zeta potential dependence on pH and ionic strength effects. Likewise, both in PBS and in SNF, a variation of the zeta potential occurs in each of these two electrolyte solutions causing the repulsive forces among the colloidal particles to exceed the attractive ones, avoiding the aggregation and determining the decrease in particle size. However, due to the pH value of the PBS (i.e., 7.4), it was not surprising to observe that the suspension turned black just because the DA autoxidation reaction occured after 17 h of incubation. Instead, under the acidic conditions of SNF (pH value 6), such DA decomposition is low and easily controlled [44]. Hence, GSE/DA-SLN in SNF can be considered as optimal condition for a nose-to-brain delivery of the neurotransmitter combined with the polyphenol antioxidant compounds not only for the reduced mean diameters of particles but also for avoiding the autoxidation reaction of the neurotransmitter. Regarding mucoadeshive studies (Figure 5a), GSE/DA SLN evidenced a noticeable mucoadeshion effect in SNF higher than DA-SLN previously studied by us [23]. To account for this result, our hypothesis is that the -OH groups of the polyphenol compounds occurring in the GSE mixture significantly contribute to the final mucoadhesive performance, giving rise to hydrogen bonding-mediated interactions with mucin macromolecules [41,42].

**Figure 8:**
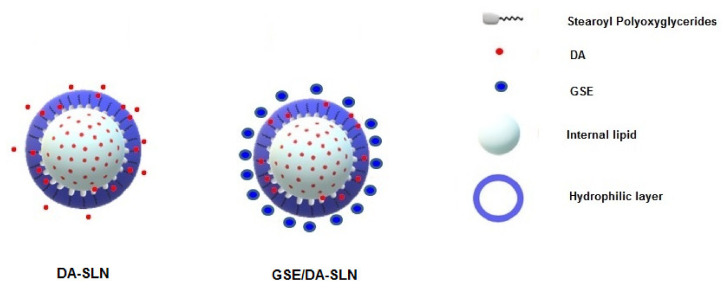
Schematic representation of SLN herein described.

Further interesting results are those observed in the in vitro release studies of GSE/DA-SLN in SNF. As shown in Figure 5b, the release of GSE from GSE/DA-SLN displayed a clear burst effect, while DA was delivered in a slower fashion. This slow DA release from GSE/DA-SLN may be advantageous for nose-to-brain delivery of the neurotransmitter because it should cause a lower neuronal toxicity consequent to a reduced stimulation of dopaminergic neurons [45]. Moreover, the finding that DA remained intact for all over the time of the study (72 h) is noteworthy, and is probably due to the simultaneous presence of the antioxidant GSE. This finding should also have a relevant implication for nose-to-brain delivery because the lack of DA decomposition for a prolonged time should limit the OS and neuroinflammation mechanisms. The results from the in vitro release studies of GSE/DA-SLN allowed us to gain insight about the localization of the species involved in Gelucire^®^ 50/13 based SLN. The burst effect observed for the release of GSE from GSE/DA-SLN suggests that the polyphenol antioxidant compounds should be localized on the surface of these nanocarriers. On the other hand, as already reported for DA-SLN [23], the neurotransmitter should be entrapped in the hydrophilic shell of polyoxyethylene chains of Gelucire^®^ 50/13 and cosurfactant (Tween 85), or within the internal lipid core of the stearoyl moieties of Gelucire^®^ 50/13 as shown in Figure 8. However, such localizations of the species involved in GSE/DA-SLN should be confirmed with further surface analyses of these nanocarriers. Similarly, understanding the mechanisms that cause the GSE localization on the surface of GSE/DA-SLN is important for achieving better control of the resulting nanocarriers but, also in this case, an in-depth study is necessary. Regardless of the exact mechanism, in our opinion, the adsorption of GSE by the surface of DA-SLN should be mainly based on a hydrogen bonding interaction between the hydroxyl groups of the polyphenol compounds represented in GSE and the polyoxyethylene chains of Gelucire^®^ 50/13 occurring in the hydrophilic shell of DA-SLN. It is noteworthy that such hydrogen bonding interactions have been invoked for a an HPLC purification method of proanthocyanidins and polyethylene glycol-coated resin in a packed column [46]. The localization of GSE on the surface of GSE/DA-SLN could also account for the satisfactory antioxidant activity measured for these particles by in vitro DPPH assay, the results of which were comparable to that of GSE-SLN.

However, in our study the GSE release profile was studied in the presence of SNF, showing a burst effect. While this method could be good enough to give early “in vitro” indications about the way by which GSE is released from GSE/DA-SLNs, it is still far from being optimal. Indeed, when SLN are introduced into a biological environment such as the nasal cavity, they should be covered by a dynamic layer of biomolecules, in particular proteins, forming the so-called “protein corona”. As a consequence of protein corona formation, SLN lose their synthetic identity and attain a new one that we can consider their “biological identity”. Protein adsorption can modify different aspects of the behavior of SLN and these changes may explain why the majority of the in vitro and in vivo studies do not correlate as expected. Moreover, the results of in vitro release in simulated fluid not containing proteins (e.g., in our case SNF) should be extrapolated with extreme care, because the “in vivo context” is difficult to simulate. Indeed, it must be taken into account that the protein corona may also affect the controlled release of drugs by the nanosystems. As described by Behzadi et al. [47] for some synthetic nanosystems and for Abraxane^®^, the formation of thick protein layers can delay the release of the nanosystem’s cargo. This may be easily explained by a steric hindrance since the protein corona represents an extra barrier between the inner part of the nanosystem and the medium. Moreover, the findings from drug release studies on GSE/DA-SLN may raise the question as to why, since GSE is completely released after five hours, only DA-SLN is delivered in the brain. In our opinion, it seems unlikely considering that it has been calculated that intranasally administered drugs should be able to reach the brain in as little as 45 min for low molecular weight substances [13] as well as within two to four hours for nanoparticulate systems [14]. Hence, it seems probable a co-delivery of GSE and GSE/DA-SLN to the brain.

From a biological viewpoint, formulations used in this study were not cytotoxic to both OECs and SH-SY5Y cells. OECs are unique glial cells found in the olfactory system that have been shown to exert direct neuroprotective effects or to be a valuable support for enhancing efficacy in cell transplantation protocols in PD [48,49,50]. In the study presented herein we have evaluated them in the context of nose-to-brain delivery of DA and GSE [51]. Human neuroblastoma cell line SH-SY5Y has been used as a suitable experimental model for studying PD from the molecular and cellular mechanism standpoints [52,53,54]. Moreover, the neurotoxin 6-OHDA has been often used in conjunction with SH-SY5Y cells as in the in vitro model for PD [32,52]. Once entered into cells, 6-OHDA triggers the formation of ROS and catecholamine quinones, leading to oxidative stress and cell death [55]. None of the SLN formulations employed at the maximal concentrations of 75 mM DA and 34.5 μg/mL grape seed pro-anthocyanidins were cytotoxic to both OECs and SH-SY5Y cells, indicating a good biocompatibility of SLN and their cargoes. On the other hand, GSE/DA-SLN and SLN carrying GSE on their surface significantly recovered the loss of SH-SY5Y cell viability determined by 6-OHDA, an effect likely due to the anti-oxidant activity exerted by GSE [25].

Among the limits of nose-to-brain delivery, it is worth mentioning the possible immunogenicity of drug vehicles and their cargos. The adjacent regions to olfactory mucosa, as the respiratory region, are highly vascularized and thus may favor a systemic uptake [56]. Nevertheless, the presence of PEG in Gelucire^®^ 50/13 may increase the half-life of SLN in the bloodstream by reducing their metabolic degradation and immunogenicity [57]. However, recent data have indicated the occurrence of immunogenic responses against PEG, resulting in hypersensitivity reactions and lack of therapeutic efficacy of the administered drug [58,59]. Finally, although immunogenicity of biopharmaceutical proteins in nose-to-brain delivery should be considered [56], in our case this event is limited by the use of biodegradable and biocompatible lipids in the SLN scaffold and of cargoes such as an endogenous catecholamine (dopamine) and the flavonoid compounds pro-anthocyanidins. Overall, further studies must consider all the aspects related to the immunogenicity of our SLN formulations in the nose-to-brain delivery scenario.

## 4. Materials and Methods

### 4.1. Materials

Grape Seed Extract containing ≥95.0% of proanthocyanidins was kindly provided by Farmalabor (Canosa di Puglia, Italy). Dopamine hydrochloride, carboxyl ester hydrolase (E.C. 3.1.1.1, 15 units/mg solid) from porcine liver, porcine stomach mucin (type II, bound sialic acid ~1%), DPPH, Tween^®^ 85 as well as the salts used for buffer preparation were purchased from Sigma-Aldrich (Milan, Italy). Gelucire^®^ 50/13 was a gift by Gattefossè (Milan, Italy). Throughout this work, double distilled water was used. All other chemicals were of reagent grade.

### 4.2. Quantitative Determination of DA and GSE

The quantitative determination of DA and GSE was carried out by HPLC as previously reported [45,60] by using a HPLC apparatus comprising a Waters Model 600 pump (Waters Corp., Milford, MA, USA), a Waters 2996 photodiode array detector and a 20 μL loop injection autosampler (Waters 717 plus). A Synergy Hydro-RP (25 cm × 4.6 mm, 4 μm particles; Phenomenex, Torrance, CA, USA) column in conjunction with a precolumn C18 insert as a stationary phase was used for analyses, and the elution of the column in isocratic mode took place at the flow rate of 0.7 mL/min. The composition of the mobile phase consisted of 0.02 M potassium phosphate buffer, pH 2.8: CH_3_OH 70:30 (*v*/*v*). Under such chromatographic conditions, retention times of DA and GSE were equal to 5.5 min and 12 min, respectively. For DA calibration, curve linearity (R^2^ > 0.999) was checked over the range of concentrations tested (4.75 × 10^−4^ to 1.5 × 10^−5^ M) and for GSE calibration curve linearity (R^2^ > 0.999) was checked over the range of concentrations equal to 100–50 μg/mL. For GSE quantification related to in vitro release studies a fluorometer apparatus (Varian Cary Eclipse, Mulgrave, Australia, excitation wavelength: 560 nm; emission wavelength: 583 nm; slits: 2.5 nm) was used and the linearity was checked over the range of concentrations equal to 50–2.5 μg/mL.

### 4.3. SLN Formulation

The preparation of DA-loaded Gelucire^®^ 50/13 SLN (DA-SLN) was made following the melt homogenization method [61]. Briefly, Gelucire^®^ 50/13 (60 mg) was melted at 70 °C and, in a separate vial, DA (10 mg), the surfactant (Tween^®^ 85, 60 mg) and 1.37 mL diluted acetic acid, 0.01%, *w*/*v*, were prepared and, then, heated at 70 °C. The resulting mixture was added to the melted phase at 70 °C in order to obtain an emulsion by homogenization at 12,300× *g* rpm for 2 min with an UltraTurrax model T25 apparatus (Janke and Kunkel, Germany). Next, the nanosuspension was cooled at room temperature and the resulting DA-SLN centrifuged (16,000× *g*, 45 min, Eppendorf 5415D, Hamburg, Germany) and the obtained pellet was re-suspended in distilled water for further studies.

Plain SLN were prepared following the same procedure above described without the addition of 10 mg DA to the aqueous phase.

To prepare GSE/DA-SLN, a suspension of DA-SLN (0.5 mL), obtained as above reported, was incubated with 1 mL of GSE aqueous solution (1 mg/mL concentration) at room temperature for 3 h under light protection and mild stirring (50 oscillations/min). Then, the mixture was centrifuged at 16,000× *g*, for 45 min (Eppendorf 5415D, Germany) and the supernatant was discarded. GSE-SLN were obtained by incubation of 0.5 mL of plain SLN previously prepared with a light protected aqueous GSE solution (1 mL volume and 1 mg/mL concentration) at room temperature for 3 h under stirring (50 oscillations/min). Then, the mixture was centrifuged at 16,000× *g* for 45 min (Eppendorf 5415D, Germany) and the supernatant was discarded.

### 4.4. Physico-Chemical Characterization of SLN

Particle size and PDI for formulations under investigations were determined by using a Zetasizer Nano ZS (ZEN 3600, Malvern, UK) apparatus according to photon correlation spectroscopy (PCS) mode. Particle size and PDI were measured after dilution 1:1 (*v*:*v*) with double distilled water, while the zeta-potential value was determined after dilution of the sample 1:20 (*v*:*v*) in the presence of KCl (1 mM, pH 7).

To determine the E.E. of DA and/or GSE in the SLN, freeze-dried particles were cleaved upon enzymatic digestion operated by carboxyl ester hydrolase. Firstly, the enzyme was dissolved at 12 I.U./mL in phosphate buffer (pH 5) and aliquots of freeze-dried SLN in the range 1–2 mg were incubated with 1 mL of the enzyme solution for 30 min in an agitated (40 rpm/min) water bath set at 37 °C (Julabo, Milan, Italy). Afterwards, samples were centrifuged (16,000× *g*, 45 min, Eppendorf 5415D) and the resulting supernatant was analyzed by HPLC for DA (or GSE) content. The E.E.% was calculated as follows:

E.E.% = DA (or GSE) in the supernatant after esterase assay/Total DA (or GSE) × 100

where total DA (or total GSE) is intended as the starting amount of neurotransmitter (or GSE) used for SLN preparation. Each measurement was performed in triplicate.

For DA-containing SLN (in the presence and absence of GSE), TEM observations were also carried out. The morphology and dimensions of DA-containing SLN (in the presence and absence of GSE), SLN were determined by cryogenic transmission electron microscopy (Cryo-TEM). All observations were performed using a Hitachi 7700 electron microscope, at a temperature of 105 K and an acceleration voltage of 100 KV. The procedure for vitrifying the samples, as previously described [62,63], can be summarized as follows. A drop of solution containing the nanocarriers was deposited on copper grids covered with an amorphous carbon film. After removing the excess solution with buffer paper, the sample was vitrified by immersion in liquid ethane maintained just above its freezing point. Then, the sample was transferred to the Gatan 626 cryo holder. The digital images were acquired with an AMT-XR-81 camera and processed with the EMIP software. Counting and size distribution of the nanoparticles were obtained by processing the obtained TEM images. Twenty fields, randomly chosen, were taken into consideration for each sample, and the morphology and particle size of the particles present in randomly selected areas on the basis of the count of 500 particles for each sample were determined.

### 4.5. Physical Stability of GSE/DA-SLN

The physical stability of GSE/DA-SLN was evaluated by measuring particle size after incubation upon the following conditions of storage: (i) 4 °C for 3 months; (ii) 25 °C for 1 week [64]; (iii) 37 °C for 24 h in phosphate buffer solution (PBS, pH 7.4); (iv) 37 °C for 24 h in Simulated Nasal Fluid (SNF, pH 6.0) [45]. SNF was prepared dissolving in water at pH 6 CaCl_2_ 2H_2_O (0.32 mg/mL), KCl (1.29 mg/mL) and NaCl (7.45 mg/mL). For each condition of storage, the particle size was measured at the time points reported in Section 2.2.

### 4.6. Solid State Study 

The solid-state study of the SLN prepared was based on FT-IR spectroscopy and thermal analysis (DSC). IR spectra were obtained in KBr discs using a Perkin Elmer 1600 FT-IR spectrometer (Perkin Elmer, Italy). The samples processed were bulk materials, DA-SLN, GSE-SLN and GSE/DA-SLN. The range examined was 4000–400 cm^−1^ with a resolution of 1 cm. DSC thermograms were obtained for bulk materials, DA-SLN and GSE/DA-SLN using a Mettler Toledo DSC 822e STARe 202 System equipped with a DSC MettlerSTARe Software. For DSC analysis, aliquots of about 5 mg of each product were placed in an aluminum pan and hermetically sealed. The scanning rate was of 5 °C/min under a nitrogen flow of 20 cm^−3^/min and the temperature range was from 25 to 275 °C for all samples. The calorimetric system was calibrated using indium (99.9%) following the procedure of the MettlerSTARe Software. Each experiment was carried out in triplicate.

### 4.7. In Vitro Mucoadhesive Studies

The mucoadhesive properties of GSE/DA- SLN were assessed in Simulated Nasal Fluid (SNF) by turbidimetric measurements [65]. SNF was prepared after dissolution of CaCl_2_ 2H_2_O (0.32 mg/mL), KCl (1.29 mg/mL) and NaCl (7.45 mg/mL) in water at pH values in the range 5–6 [66].

To 6 mL of freshly prepared mucin dispersions in SNF (1 mg/mL) held in a water bath (Julabo, Milan, Italy) at 37 °C under stirring (150 rpm), freeze-dried GSE/DA-SLN, previously dispersed in 6 mL of SNF, were added. The turbidity of the stirred mixture at 37 °C was measured at 0, 2, 5, 7 and 24 h at the wavelength of 650 nm using a Perkin-Elmer Lambda Bio 20 spectrophotometer. HEC dissolved in SNF (0.4 mg/mL) was taken as a positive control. Each experiment was performed in triplicate and the results are expressed as mean ± standard deviation of each mean.

### 4.8. In Vitro Release Studies

Freeze-dried pellets of GSE/DA-SLN (corresponding to 2–3 mg of DA and 1–1.5 mg GSE) were dispersed in 20 mL of SNF containing 1 mg of carboxyl ester hydrolase and thermostated at 37 ± 0.1 °C in an agitated (40 rpm/min) water bath (Julabo, Milan, Italy). At scheduled time-points, from the receiving medium 0.5 mL were withdrawn and replaced with 0.5 mL of fresh medium. Each sample was centrifuged (16,000× *g*, 45 min, Eppendorf 5415D, Germany), and the concentrations of DA and GSE were determined in the resulting supernatants by HPLC or HPLC/fluorometry as above described, respectively. For the cumulative release calculation, the approach reported in the literature was followed [67]. All release experiments were carried out in triplicate.

### 4.9. Antioxidant Activity of Starting Materials and SLN 

The in vitro antioxidant activity of bulk materials, plain SLN, DA-SLN, GSE-SLN and GSE/DA-SLN were evaluated using the DPPH test with slight modifications [31]. Briefly, the DPPH was dissolved in ethanol to obtain a stock solution at the concentration of 0.001% *w*/*v* and, then, diluted to 8 × 10^−4^% (*w*/*v*). After the freeze-drying of SLN, 0.5 mL of each sample previously re-dispersed in ethanol was reacted with 2.5 mL of the diluted DPPH solution for 60 min at room temperature in the dark. The corresponding absorbances were recorded at the wavelength of 514 nm. Blanks were obtained by filling the cuvettes with 0.5 mL of ethanol and 2.5 mL of the 8 × 10^−4^% (*w*/*v*) solution in DPPH. The control GSE was prepared by dissolving the powder in water at the concentration of 2 mg/mL and 50 μL were mixed with 0.45 mL of ethanol and then incubated as above with 2.5 mL of DPPH test reagent. Pure Gelucire^®^ 50/13 was dispersed in acetone at 5 mg/mL by vortexing and, afterwards, treated as above with DPPH reactant. For all formulations under investigation, antioxidant activity (AA) was calculated from Equation (1) and expressed in percentages:

AA (%) = (1 − As/Ab) × 100(1)

where As is the sample absorbance, and Ab the absorbance of the radical.

### 4.10. In Vitro Cytotoxicity Studies of SLN

OECs or SH-SY5Y cells were plated at the number of 30,000 and 40,000 per each well of a 96-well plate, respectively. Cells were incubated with either no GSE/DA-SLN (200 μL, 66 μL, 50 μL containing respectively 16.0, 5.3 and 4 μg/mL GSE), GSE/DA-SLN (200 μL, 66 μL, 50 μL of an initial stock solution of 75 μM DA and 34.5 μg/mL GSE), DA-SLN (200 μL, 66 μL, 50 μL of an initial stock solution of 75 μM DA) and plain SLN ((200 μL, 66 μL, 50 μL) in complete medium (final volume per well was 200 μL). After 24 h, cells were tested for viability by the MTT assay. Briefly, a stock solution of MTT (Sigma) in phosphate-buffered saline (PBS) (5 mg/mL) was added to each well reaching a final concentration of 0.5 mg/mL. After 4 h, the formazan crystals were dissolved in DMSO and measured spectrophotometrically by a microplate reader (PowerWave HT, Bio-tek, Milan, Italy) at a wavelength of 595 nm. The relative viability was calculated with respect to control wells containing mock cells, i.e., cells treated with PBS (considered as 100%). 0.1% Triton-X100-treated cells were used as a positive control.

### 4.11. Cytoprotective Effects of SLN

SH-SY5Y were plated for 24 h on plastic culture plates at a density of 40,000 cells per well (96-well plates). The cytoprotective effect of GSE/SLN-DA on 6-OHDA-induced SH-SY5Y cells was measured with an MTT-based colorimetric assay, as specified above. Cells were co-treated with a defined concentration of 6-OHDA (100 μM), GSE/DA-SLN (200 μL of a solution of 75 μM DA) or SLN-GSE (200 μL of a solution of 16 μg/mL) for 24 h. As before, the final volume per well was 200 μL. MTT solution was added at the end of the treatment to the cell culture media at 0.5 mg/mL final concentration and incubated for 4 h at 37 °C in the dark. The absorbance at 595 nm was determined with a microplate reader. The concentration of 6-OHDA used for incubation with cells was chosen based on preliminary experiments that evaluated the range in which was observed an overt reduced viability, as assessed by the MTT method. H_2_O_2_ (100 μM) was used as a positive control.

### 4.12. Statistics

Statistical analyses were carried out by Prism v. 4, GraphPad Software Inc., San Diego, CA, USA. Data were expressed as either mean ± SD. Multiple comparisons were based on one-way analysis of variance (ANOVA) together with Bonferroni’s or Tukey’s post hoc test and differences were considered significant when *p* < 0.05.

## 5. Conclusions

Nose-to-brain delivery of DA in combination with the antioxidant grape seed-derived polyphenol compounds is feasible and advantageous using Gelucire^®^ 50/13-based SLN as demonstrated by the findings of the present work. In fact, GSE/DA-SLN, possessing reduced mean diameters in SNF and being able to avoid the autoxidation reaction of the neurotransmitter for a prolonged time as well as releasing DA in a sustained manner, bringing about a lower neuronal toxicity, may be considered promising system for brain-targeted delivery of the neurotransmitter by intranasal administration. Furthermore, their interest is even fueled by their cytocompatibility and dampening of cell cytotoxicity exerted by a well-known neurotoxin. This platform of colloidal carriers may be considered an advantageous alternative to the pro- or co-drug chemical approach to overcome the BBB obstacle by the neurotransmitter [68]. Further investigations are currently ongoing, in order to assess the in vivo performances of these vehicles for novel approaches to be applied to PD treatment.

## Figures and Tables

**Table 1:** Physico-chemical Characterization of SLN.

Formulation (Code)	Particle Size (nm)	Polydispersity Index (PDI)	Zeta Potential (mV)	Encapsulation Efficiency (E.E., %)	pH	Ref.
Plain SLN	141 ± 11	0.34 ± 0.06	−9.7 ± 0.8		3.4 ± 0.07	[21]
DA-SLN	171 ± 6	0.20 ± 0.01	−2.0 ± 0.7	19 ± 3 (DA)	3.1 ± 0.02	[22]
GSE-SLN	118 ± 6	0.20 ± 0.02	−7.3 ± 1.1	65 ± 3 (GSE)	3.7 ± 0.00	
GSE/DA-SLN	184 ± 34	0.32 ± 0.07	−2.7 ± 0.2	14 ± 1 (DA), 54 ±8 (GSE)	3.3 ± 0.03	

**Table 2:** Antioxidant Activity of Some Starting Materials and SLN.

Formulation (Code)	Antioxidant Activity (%)
Pure GSE	100.0 ± 0.6
Pure Gelucire^®^ 50/13	55.0 ± 0.2
Plain SLN	72.8 ± 5.3
DA-SLN	54.7 ± 2.5
GSE-SLN	84.3 ± 3.3
GSE/DA-SLN	78.6 ± 3.6
